# An *In Vitro* Diagnostic for Multiple Sclerosis Based on C-peptide Binding to Erythrocytes

**DOI:** 10.1016/j.ebiom.2016.07.036

**Published:** 2016-08-04

**Authors:** Sarah Y. Lockwood, Suzanne Summers, Eric Eggenberger, Dana M. Spence

**Affiliations:** a Department of Chemistry; b Department of Neurology; c Department of Cell & Molecular Biology

**Keywords:** Multiple Sclerosis, Diagnostic, C-peptide, Erythrocytes

## Abstract

**Objective:**

To investigate the utility of a blood-based lab test as an aid in identifying patients with Multiple Sclerosis (MS).

**Methods:**

Whole blood from subjects with MS, non-MS neurologic diseases, and healthy controls was centrifuged to isolate erythrocytes. Following the addition of exogenous C-peptide, the supernatant was assayed for remaining C-peptide using an enzyme linked immunosorbent assay (ELISA).

**Results:**

The cohort included subjects with MS (n = 86), other non-MS neurologic diseases (OND n = 75), and healthy controls (n = 39). The average C-peptide bound to erythrocytes in MS samples (3.51 ± 0.59 pmol) was significantly higher than non-MS subjects (2.23 ± 0.51 pmol; p < 0.001) and healthy controls (1.99 ± 0.32 pmol; p < 0.001). Using a cutoff of 3.04 pmol of C-peptide uptake, the test exhibited a sensitivity of 98.3% and specificity of 89.5%. A receiver-operator characteristic (ROC) curve generated from the ratio of the sensitivity to 1-selectivity resulted in an area under the curve of 0.97.

**Conclusions:**

Exogenous C-peptide binding to erythrocytes has potential value in distinguishing MS subjects from non-MS neurologic diseases and healthy controls.

## Highlights

• A blood-based diagnostic for Multiple Sclerosis is reported.
• Based on exogenous C-peptide binding to harvested red cells
• Results are independent of age, disease duration, therapies.

Biomarkers and point of care diagnostics are lacking in Multiple Sclerosis (MS), despite hallmark features often found in many diagnosed patients. Efforts to determine causes of this breakdown are ongoing by many groups. Here, we report that the addition of a molecule that is naturally occurring in most humans to a sample of blood obtained in a simple blood draw, may serve as a fast and simple auxiliary test during the diagnosing stage of the disease.

## 1 Introduction

It is estimated by the National Multiple Sclerosis Society that over 2.3 million people worldwide have Multiple Sclerosis (MS) and that approximately 250,000 to 350,000 live in the United States. It is also estimated that nearly 10,000 new cases are diagnosed each year in the United States and the rate of diagnosed cases is increasing. Unfortunately, the cause of MS and its rate of increase are unknown. There is no cure for MS, although some therapies are available for those people with the disease.

The diagnosis of MS is often challenging, causing frequent errors or delays (Solomon et al., 2016). There is no single test that is performed to diagnose the disease; rather, the diagnostic process involves physical and neurological examinations, multiple MRIs, and a 6-month to 2-year time frame prior to final diagnosis, (Polman et al., 2011) which even then may be a “soft” diagnosis. Collectively, the exhaustive process of diagnosing (or ruling out) MS is costly and can adversely affect the patient as MS therapies may delayed during the diagnosing process.

A simple, objective bloodstream-based biomarker remains an unmet clinical need (Polman et al., 2011). There have been recent reports of blood-based diagnostics in the literature, although these tests have suffered from either small patient numbers or very low diagnostic sensitivity. Here, we evaluate the performance of a blood-based lab test in distinguishing subjects with MS compared to subjects with other, non-MS neurologic disease (ONDs) and healthy controls. Specifically, we determined the amount of exogenously added C-peptide bound to a sample of erythrocytes (ERYs) obtained from the whole blood of people with MS to ERYs of healthy controls and controls with ONDs.

## 2 Methods

The institutional review board at Michigan State University approved all study protocols, and written informed consent was obtained from all participants. Subjects were recruited from the Michigan State University Department of Neurology and Ophthalmology Clinic, and comprised consecutive MS patients meeting McDonald criteria, or ONDs. A convenience sample of primarily lab personnel served as healthy controls.

Approximately 7.5 mL of whole blood was obtained by venipuncture into citrate-coated tubes under vacuum. All whole blood samples were processed within 12 h after the blood draw. Processing started by centrifuging the whole blood at 500*g*, and after removal of the buffy layer by aspiration, the ERYs were washed with physiological salt solution (PSS) prior to determination of hematocrit using a hematocrit centrifuge analyzer. Crude C-peptide was purified by high performance liquid chromatography; 100% purity was confirmed by mass spectrometry and used to prepare 15 mL of a stock solution of approximately 8 μM C-peptide in distilled and deionized water (concentration verified using a commercial ELISA kit). On the same day as an analysis of C-peptide binding, a working solution of C-peptide (800 nM) was prepared by diluting 100 μL of the 8 μM stock solution to 1 mL with distilled and deionized water (DDW).

20 pmol of C-peptide (25 μL of the 800 nM solution) were added to approximately 900 μL of PSS, followed by the immediate addition of an aliquot of the purified ERYs (~ 100 μL, although amounts vary based on hematocrit of the packed, purified ERYs) to result in a 7% solution of ERYs in the final C-peptide containing sample. After 2 h of incubation at 37 °C, the sample was centrifuged at 500*g*. An aliquot of the supernatant above the packed ERYs, as well as all standards, was then diluted 1:50 in DDW and used as the sample in an ELISA for C-peptide. The amount of C-peptide remaining in the supernatant was measured, and by subtracting this amount from the 20 pmol originally added to the sample, the amount bound to the ERYs was calculated. C-peptide standards were prepared in PSS, diluted, and measured in the same manner as the samples for quantitation, save for the addition of the ERYs.

Thus, it is important to note that all samples were treated in an identical manner after the initial centrifugation of the whole blood. Incubation times and temperatures were identical for all samples (MS, OND, and controls); calibrations were performed with standard C-peptide solutions prior to analysis of samples.

The average C-peptide ± standard deviation is reported for all patient groups and statistical significance is reported for all groups using Student's *t*-test with associated p-values.

## 3 Results

### 3.1 Participant Data

Table 1 shows a breakdown of the 200 participants, 86 subjects had MS (68% female, average age of 52 years, average disease duration 14.1 years, 76% treated with disease modifying therapies). Participants with ONDs comprised 75 of the participants (46% female, average age 63 years). The remaining 39 study participants were healthy controls (54% female, average age of 36 years).

### 3.2 C-peptide Binding to ERYs

**Fig. 1:**
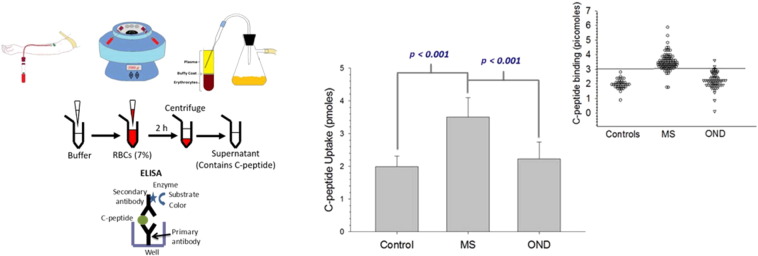
Mean C-peptide uptake for control cohorts and MS patients. Approximately 7 mL of whole blood is drawn by venipuncture, followed by centrifugation to pellet the ERYs. An aliquot of these ERYs are added to a buffer that contains albumin and C-peptide. After an incubation period, the ERYs are centrifuged and the supernatant is analyzed for remaining, free C-peptide *via* ELISA. The amount of C-peptide bound to the ERYs is obtained by subtracting the amount of C-peptide in the supernatant from the C-peptide originally added (20 pmol). The mean of each data set is shown in the bar graph in the middle. The ERYs from the healthy controls (n = 86) had an average C-peptide binding of 1.99 ± 0.32 pmol. The ERYs from the ONDs (n = 75) bound an average of 2.23 ± 0.51 pmol. The ERYs from MS patients (n = 53) bound a significantly higher amount (p < 0.001) than both the healthy and non-MS neurological controls, with an average of 3.51 ± 0.0.59 pmol. Error bars represent standard deviations. The smaller dot plot on the right is presented in order to show the individual C-peptide binding values for each participant.

The average binding of C-peptide to ERYs was 3.51 ± 0.59 pmol in MS subjects (n = 86), higher than the 2.23 ± 0.51 pmol in subjects with ONDs (n = 75; p < 0.001), or the 1.99 ± 0.32 pmol in healthy control (n = 39; p < 0.001), all statistically different (p < 0.001); (Fig. 1). Among the subcohort of subjects with ONDs, the subjects with myasthenia gravis (n = 7) demonstrated a tight cluster of elevated C-peptide binding of 2.72 ± 0.31 pmol; when these subjects are removed from the data set, the average binding for the ONDs is 2.16 ± 0.52 pmol with greater separation from the MS cohort.

**Fig. 2:**
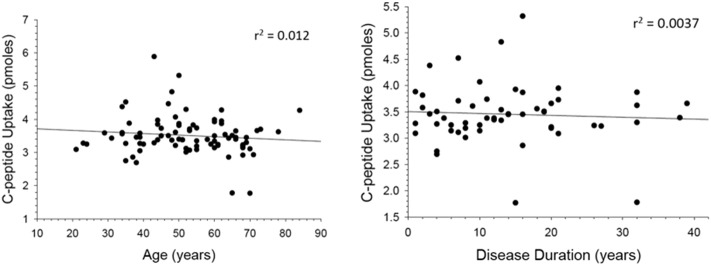
C-peptide uptake for MS patients based on age of subject and disease duration. C-peptide uptake was analyzed based on the age of the MS subject (n = 84 subjects reporting, left figure) or the duration of the disease (n = 54 subjects reporting, right figure). The correlation, provided by the values of coefficient of determination within the figures, was not statistically different from zero at 95%.

While the data reported in this study is well-powered, a weakness may be in the age differences between the controls and the MS patients. Therefore, we evaluated the C-peptide uptake results as a function of subject age and duration of disease. These results, shown in Fig. 2, clearly show there is no correlation between C-peptide uptake by the cells and subject age or how long the subject has had MS.

In addition to age and disease duration, data was also evaluated based on any disease modifying therapies administered to the MS patients. These data were obtained for the 86 patients with MS and, as shown in Table 2 below, there was no significant difference between the 16 patients with MS on no disease modifying therapies and the remaining 70 patients on some form of modifying therapy.

### 3.3 Diagnostic Evaluation of C-peptide Uptake

**Fig. 3:**
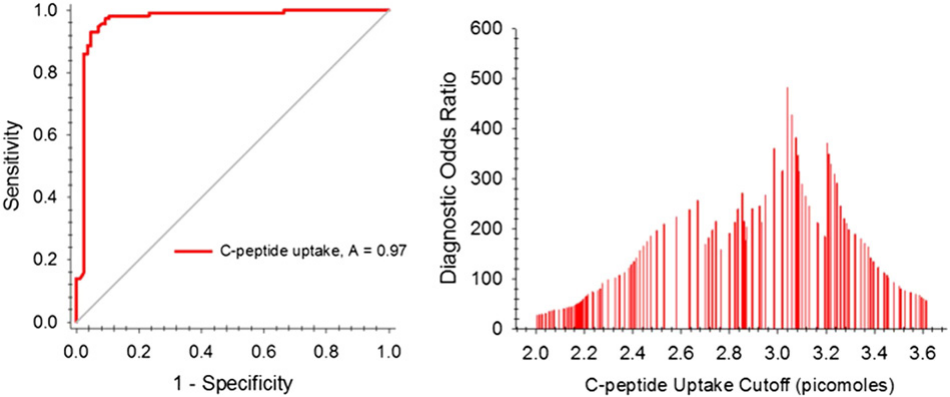
Evaluation of C-peptide uptake as a potential diagnostic tool. An ROC curve showing the true positive rate (sensitivity) as a function of the false positive rate (1-specificity) is shown on the left. The area under the curve for this curve is 0.97; typically, a useful diagnostic has an AUC of about 0.80. The data on the right represent various diagnostic odds ratios (DOR) at C-peptide uptake values. The maximum DOR occurred at an uptake value of 3.04 pmol of C-peptide; at this uptake value, the sensitivity and specificity of the test were 98.3% and 89.5%, respectively.

The potential for C-peptide binding to ERYs as an *in vitro* diagnostic is strengthened upon evaluation of common diagnostic analysis tools. A receiver-operating characteristic (ROC) curve, a plot of the true positive rate as a function of false positive rate, was constructed and is shown in Fig. 3. Here, ROC curves can be used to determine C-peptide binding thresholds resulting in the highest diagnostic odds ratio (DOR). The DOR is derived from positive and negative likelihood ratios; the positive likelihood ratio is calculated as the sensitivity/(1-specificity) and the negative likelihood ratio is the specificity/(1-sensitivity). The DOR is the quotient of likelihood ratios at various C-peptide binding values. For our test, the optimum DOR (481) occurred when using 3.04 pmol of C-peptide as the threshold value between predicting whether or not a patient had MS. At this value of 3.04, the sensitivity was 98.3% and the specificity was 89.5%.

## 4 Discussion

C-peptide is secreted in equal amounts with insulin from pancreatic β-cell granules *in vivo*. Since its discovery in the late 1960’s, (Rubenstein et al., 1969) C-peptide has been regarded as a biologically inactive species once secreted from the β-cell granules (Luzi et al., 2007). In fact, other than facilitating the insulin production process, many believe C-peptide to be useful only as a biomarker for insulin production in patients with type 1 diabetes, due in large part to its longer half-life in the bloodstream (~ 30 min) in comparison to insulin (< 5 min). Furthermore, no ERY C-peptide receptor has been identified, and the mechanism of ERY C-peptide uptake remains unknown.

However, since the mid-1990’s, there have been numerous studies reporting beneficial effects of C-peptide replacement therapy to animals and humans with type 1 diabetes (Nordquist et al., 2008, Sima, 2003, Sima and Kamiya, 2004, Wahren et al., 2007). Many of these cellular and tissue effects involve improvements in blood flow (Forst et al., 2000, Forst and Kunt, 2004, Kunt et al., 1999). These studies reporting an improvement of overall blood flow inspired our group to investigate and report that C-peptide enhances the ability of ERYs to release adenosine triphosphate (ATP), a well-established stimuli of the potent vessel dilator and mediator of blood flow, nitric oxide (NO). During the course of our studies, we also reported that the ability of C-peptide to stimulate increased ERY-derived ATP also required zinc and serum albumin (Meyer et al., 2008, Liu et al., 2015). While C-peptide binds to the cell in the presence of albumin, there are no biological effects on the cell unless zinc is delivered along with the C-peptide (Liu et al., 2015). In fact, the ratio of C-peptide to zinc delivery to the cells is 1:1. Recently, others have shown enhanced effects of C-peptide if supplemented with zinc prior to *in vivo* delivery (Slinko et al., 2014).

Although we are investigating potential links between these findings and currently hypothesized models of MS pathobiology, ERY studies in MS patients are not without precedence. A report in the 1960s (Raczkiewicz and Leyko, 1966) documented enhanced production of “adenine nucleotides” from ERYs of MS patients after an oral glucose challenge, and we reported an increase in ATP release from the ERYs of MS patients *versus* healthy controls (Letourneau et al., 2010). In accordance with zinc, Dore-Duffy et al. demonstrated that MS ERY membranes have a higher zinc to cholesterol ratio than control patients (Ho et al., 1986, Dore-Duffy et al., 1983). Reports of normal plasma insulin levels of people with MS suggest that insulin and C-peptide secretions from the pancreatic β-cells of people with MS are not elevated. Collectively, it was hypothesized that the increased production of the adenine nucleotides and the increased membrane zinc levels may be due to increased delivery of zinc to the MS ERY by C-peptide.

ERY C-peptide binding in the cohort of MS subjects was significantly higher than either the ONDs (p < 0.025) or healthy controls. To date, we have analyzed 75 patients with non-MS neurologic diseases including leukodystrophy, myasthenia gravis (MG), optic neuropathy, and Parkinson's disease. While the average erythrocyte C-peptide binding of the OND subjects was higher than the healthy controls (p < 0.025), it remained significantly lower than the MS subjects. Using a cutoff of 3.04 pmol of C-peptide binding, the sensitivity was 98.3% with a specificity of 89.5%. C-peptide uptake was independent of age, disease duration, or disease-modifying therapy; there was also no statistical difference in C-peptide uptake between MS patients on a disease modifying therapy and MS patients who were not on a therapy.

The subcohort with myasthenia gravis MG (n = 7) is small, but, intriguingly, demonstrated higher C-peptide binding than the other OND subjects (p < 0.01); excluding MG subjects from this subcohort produced greater separation from the MS subjects. Due to C-peptide's association with diabetes, it should also be noted that, within our data set, 4 of the participants (1 control, 3 OND) had type 2 diabetes. While they comprise a small percentage of our 200 patient study, the average C-peptide uptake for these patients was < 1 pmol. More measurements are needed to reach meaningful conclusions, especially evaluating patients with both diabetes and MS (a rare but sometimes presenting condition), but preliminarily, the ERYs of patients with diabetes alone appear to bind less C-peptide, and therefore would not result in false positives in our test.

Further investigation using this methodology among subjects with MS accounting for such variables as MRI and clinical status, in addition to other neurologic, autoimmune, and inflammatory diseases is ongoing. In addition, validation studies will also be required if this test is to be offered as an approved *in vitro* diagnostic. We are currently working with regulatory agencies on the design and implementation of a larger validation study. This pilot study demonstrates the potential of C-peptide binding to ERYs as a possible test to assist in the diagnosis of MS, and has the advantages of being minimally invasive, employing widely available reagents, and rapid return of results.

## Funding Sources

NIH grant # EB016379-01A.

## Conflicts of Interest

Sarah Y. Lockwood: Holds stock options in Lifeblood, LLC.

Suzanne Summers: Holds stock options in Lifeblood, LLC.

Eric Eggenberger: Holds stock options in Lifeblood, LLC.

Dana M. Spence: Received honoraria for consulting visit to Abbvie, Inc., holds stock options in Lifeblood, LLC, and is supported by NIH grant #'s EB016379-01A1 and R01GM110406-01.

LifeBlood, LLC did not participate in study design, data collection, data analysis, interpretation, writing of the report.

## Author Contributions

Sarah Y. Lockwood, patient consent, analysis and interpretation of data.

Suzanne Summers, acquisition of data, analysis and interpretation of data.

Eric Eggenberger, study design, interpretation of data, study supervision, critical revision of manuscript for intellectual content.

Dana M. Spence, study concept and design, analysis and interpretation of data, study supervision, critical revision of manuscript for intellectual content.

Co-investigators: None.

## Figures and Tables

**Table 1:** Participant information and C-peptide uptake within each cohort. The values in parentheses are represent one standard deviation about the mean.

Characteristic	Total (n = 200)	Control (n = 39)	MS (n = 86)	ONDs (n = 75)
Age, y	52.9 (17.4)	35.9 (16.3)	52.2 (13.5)	62.6 (15.0)
Female	63%	53.8%	75.6%	53.3%
C-peptide binding		1.99 (0.32)	3.51 (0.59)	2.23 (0.51)
p-Value to MS		p < 0.001		p < 0.001

**Table 2:** Participant information and C-peptide uptake among the 86 MS patients based on disease modifying therapy.

	None	Glatiramer acetate	Interferon	Fingolimod	Teriflunomide	Natalizumab	Dalfampridine	Dimethyl fumarate
Uptake average	3.46	3.48	3.64	3.55	3.72	3.59	3.38	3.41
Std dev.	0.43	0.63	0.80	0.40	0.32	0.26	0.29	0.28
N	16	24	21	2	5	5	3	10
%	18.6%	28.0%	24.4%	2.2%	5.4%	5.4%	3.2%	11.8%
